# Self-adjuvanting cancer nanovaccines

**DOI:** 10.1186/s12951-022-01545-z

**Published:** 2022-07-26

**Authors:** Zhiyun Liao, Jing Huang, Pui-Chi Lo, Jonathan F. Lovell, Honglin Jin, Kunyu Yang

**Affiliations:** 1 Cancer Center, Union Hospital, Tongji Medical College, Huazhong University of Science and Technology, Wuhan, 430022 China; 2 College of Biomedicine and Health and College of Life Science and Technology, Huazhong Agricultural University, Wuhan, 430070 China; 3 Department of Biomedical Sciences, City University of Hong Kong, Tat Chee Avenue, Kowloon, Hong Kong, China; 4 Department of Biomedical Engineering, University at Buffalo, State University of New York, Buffalo, NY 14260 USA

**Keywords:** Nanovaccine, Self-adjuvanting, Cancer immunotherapy, Antigen presentation, Lymph node

## Abstract

Nanovaccines, a new generation of vaccines that use nanoparticles as carriers and/or adjuvants, have been widely used in the prevention and treatment of various diseases, including cancer. Nanovaccines have sparked considerable interest in cancer therapy due to a variety of advantages, including improved access to lymph nodes (LN), optimal packing and presentation of antigens, and induction of a persistent anti-tumor immune response. As a delivery system for cancer vaccines, various types of nanoparticles have been designed to facilitate the delivery of antigens and adjuvants to lymphoid organs and antigen-presenting cells (APCs). Particularly, some types of nanoparticles are able to confer an immune-enhancing capability and can themselves be utilized for adjuvant-like effect for vaccines, suggesting a direction for a better use of nanomaterials and the optimization of cancer vaccines. However, this role of nanoparticles in vaccines has not been well studied. To further elucidate the role of self-adjuvanting nanovaccines in cancer therapy, we review the mechanisms of antitumor vaccine adjuvants with respect to nanovaccines with self-adjuvanting properties, including enhancing cross-presentation, targeting signaling pathways, biomimicking of the natural invasion process of pathogens, and further unknown mechanisms. We surveyed self-adjuvanting cancer nanovaccines in clinical research and discussed their advantages and challenges. In this review, we classified self-adjuvanting cancer nanovaccines according to the underlying immunomodulatory mechanism, which may provide mechanistic insights into the design of nanovaccines in the future.

**Graphical Abstract:**

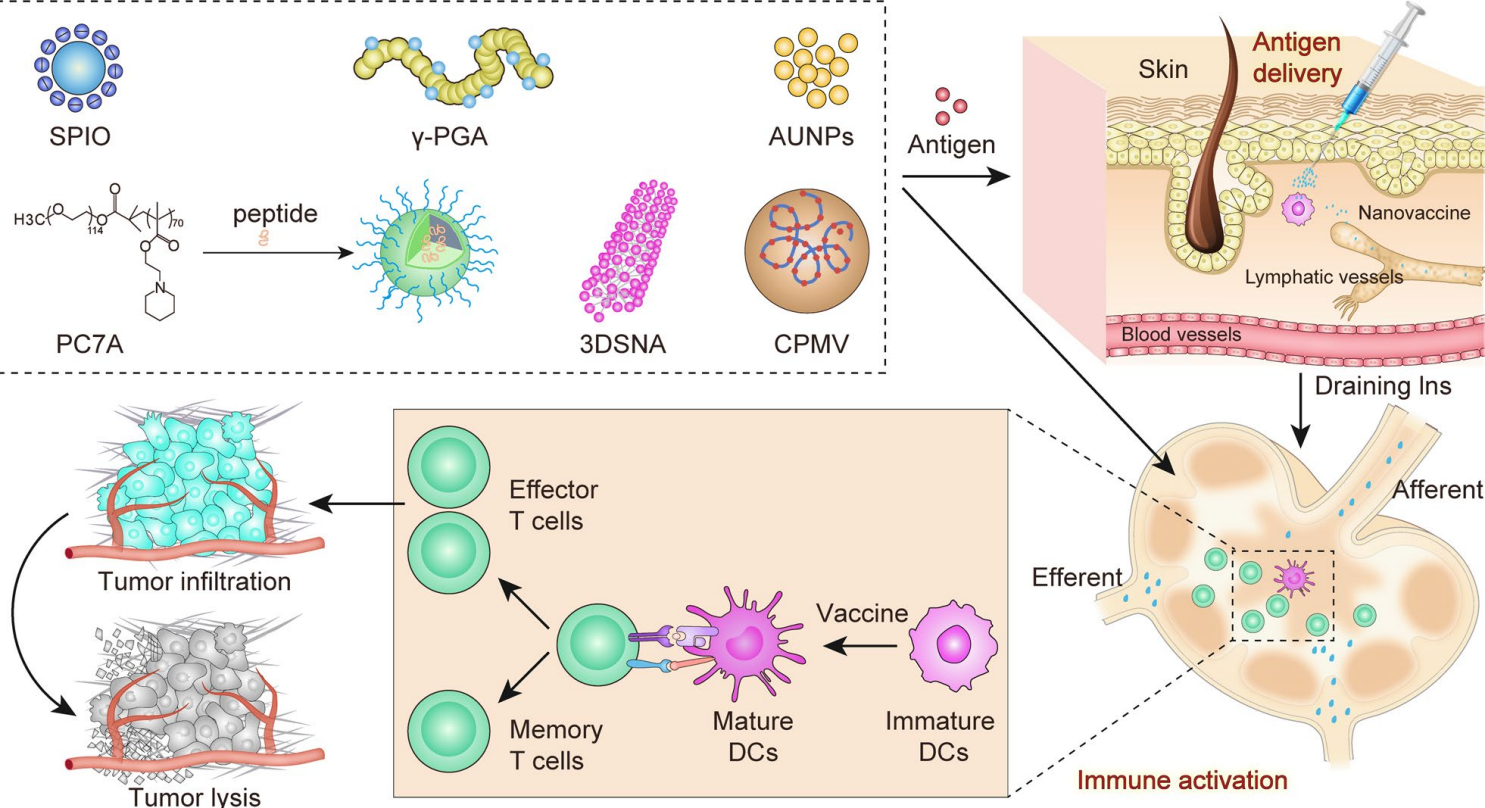

## Introduction

With the successful identification of tumor antigens, personalized neoantigen vaccines and immune checkpoint inhibitors that can reverse tumor-induced immune exhaustion, cancer vaccines have re-emerged as a promising tool for cancer immunotherapy [1]. Cancer vaccines are active immunotherapies using tumor cells, exosomes, peptides, proteins, and/or nucleic acid sequences that contain tumor-specific antigens (TSA) or tumor-associated antigens (TAA) to induce a specific immune response and eventually inhibit tumor growth [2]. The development of an efficacious vaccine against the viral pathogen severe acute respiratory syndrome coronavirus-2 (SARS-CoV-2) was unprecedented in terms of scale and speed, which has further accelerated the development of cancer vaccines [3, 4]. BioNTech-Pfizer and Moderna’s mRNA vaccine are based on lipid nanoparticles (LNP) without an exogenous traditional vaccine adjuvant, showcasing the potential for new approaches to develop effective vaccines [5]. Cancer vaccines have been explored as a potentially promising cancer treatment strategy with broad prospects for clinical application [6, 7].

The addition of adjuvants into the vaccine components is to enhance the strength, breadth, and durability of the immune response induced by them [8, 9]. Aluminium was the first adjuvant discovered empirically and is now widely considered and used for vaccine development [10]. Aluminum facilitates the formation of antigen depots, enhances antigen transport, and promotes the antigen uptake and presentation by macrophages, and preferentially induces Th2 cells, effectively activates inflammatory signals and immunity [11]. Oil-in-water emulsions are another example of an empirically validated adjuvant that promotes the antigen uptake by dendritic cells (DCs), and provides danger signals by inducing the release of ATP [12]. Aluminum based adjuvants and oil-in-water emulsion based adjuvants, like MF59, and also liposomal adjuvants like AS01 have been licensed for human vaccines [13]. In cancer vaccines, polyriboinosinic–polyribocytidylic acid [poly(I:C)] and its derivative poly-ICLC which are synthetic mimics of viral dsRNA polymers, are often used as potent adjuvants [14, 15].

Although adjuvants have been examined, the clinical transformation of cancer vaccines still faces many obstacles, including the highly immunosuppressive tumor microenvironment, down-regulation of major histocompatibility complex (MHC) class I (MHC-I) on cancer cells, ineffective activation of APCs, and inability to activate antitumor immunity [16]. Advances in nanotechnology have led to the development of nanovaccines that not only can overcome the drawbacks of traditional vaccines, but also possess advanced modulation abilities [17]. As a vaccine delivery system, nanoparticles can achieve superior efficacy for several reasons: (1) Nanoparticles can trigger tumor antigen release in situ to enhance immune response and load antigen and adjuvant simultaneously to effectively activate APCs, avoiding immune tolerance caused by immature APCs directly phagocytizing antigen [18, 19]. (2) The use of nanoparticles often offers better spatial and temporal delivery of vaccines [20]. Vaccine accumulation in LN can be significantly enhanced by manipulating the size, charge and other physical and chemical features of nanoparticles [21, 22]. Compared with free antigen, nanoparticles with sizes between 20 and 100 nm can be absorbed and retained within lymphatics [23]. As well as size, efficient LN accumulation and APCs uptake can be achieved by adjusting the surface charge [24]. Furthermore, LN stromal and immune cells, especially APCs, are essential for inducing certain types of adaptive immune response. Through the surface chemical decoration of nanoparticles, mannosylated nanovaccines achieve APC-targeting and cross-presentation capacity [25]. (3) Nanoparticles provide different administration methods, including subcutaneous administration, intranasal administration and oral administration [26]. It is worth noting that different administration methods face different immune environments. (4) Adjuvants can be delivered more precisely and with greater stability using nanomedicine platform [27].

In addition to serving as a delivery system, nanoparticles also have the ability to trigger an array of immune response, and can themselves be used as adjuvants of vaccines [28]. Herein, we consider self-adjuvanting nanovaccines, to be comprised of nanomaterials with intrinsic immunostimulatory activity that may not need the use of additional adjuvant, or at least can minimize the dosage of additional adjuvants. This self-adjuvanting property of nanovaccines for cancer therapy has been reported but not yet been well explored. In this review, we focus on the mechanisms of existing adjuvants and self-adjuvanting nanovaccines for cancer therapy, self-adjuvanting nanovaccines in clinical research, and the advantages and challenges of self-adjuvanting nanovaccines in cancer therapy.

## Mechanism of vaccine adjuvants

**Fig. 1:**
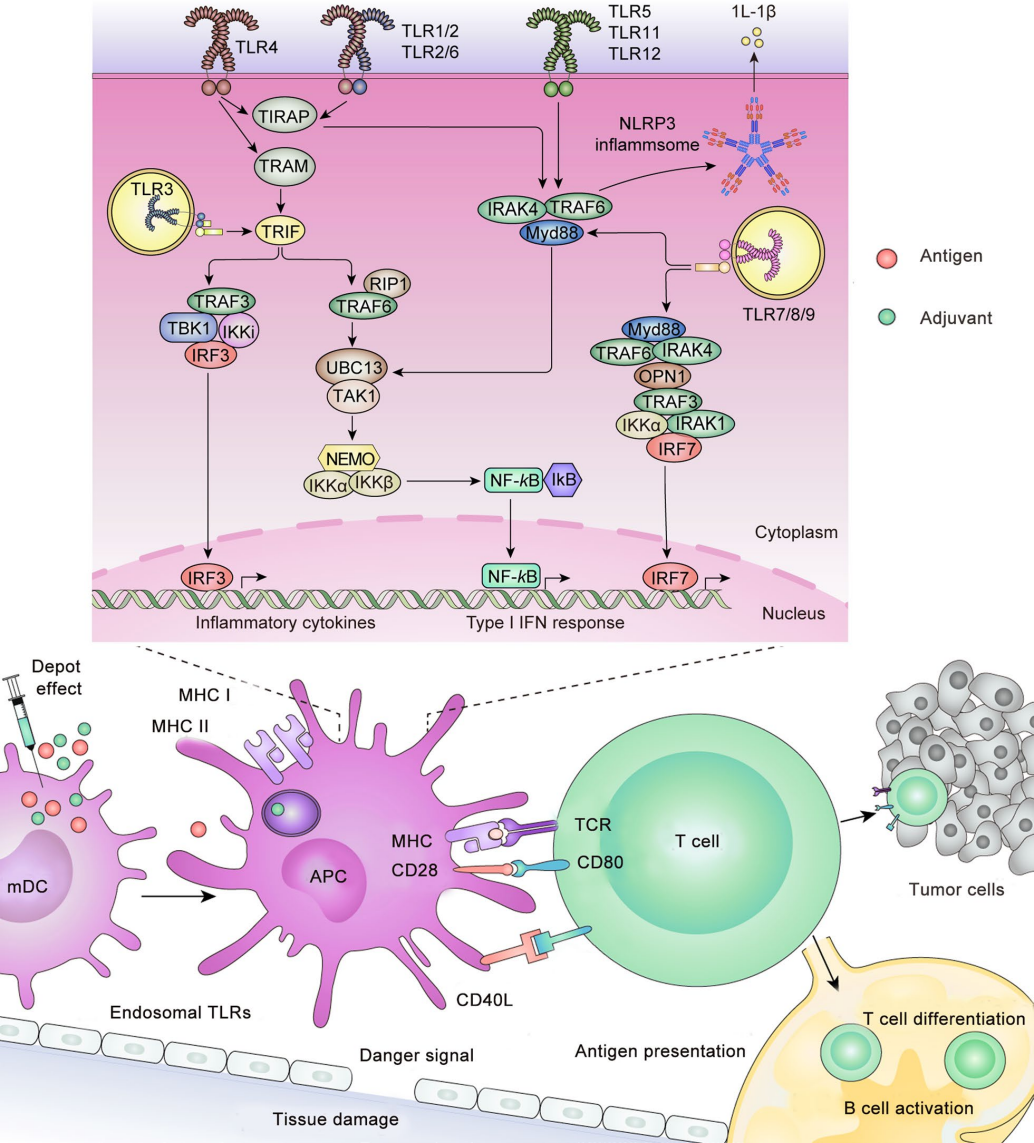
Signaling pathways in vaccine adjuvant-activated APCs. *APC* antigen presenting cells; *TLR* Toll-like receptors; *MyD88* myeloid differentiation factor 88; *TIRAP* Toll-interleukin receptor (TIR) domain containing adaptor protein; *TRAM* TIR-domain-containing adaptor-inducing interferon-β (TRIF); and TRIF-related adaptor molecule

Schijns classified the mechanism of adjuvants according to immunological concepts and defined five categories of adjuvants (Fig. 1): (1) adjuvants that facilitate antigen uptake, transport and presentation by APCs; (2) adjuvants that show a depot effect of antigen depot formation and prolonged antigen delivery; (3) adjuvants that target the pattern recognition receptor (PRR) to activate an inherent immune response; (4) adjuvants that promote APC polarization, T cell differentiation and B cell activation; (5) adjuvants that provide the danger signal of tissue damage or increased stress [29].

Adjuvants that target PRR, such as Toll-like receptors (TLRs), NOD-like receptors (NLRs), and RIG-I-like receptors (RLRs), have already been widely applied [30]. TLRs located on the surface of APC (TLR2, TLR4, TLR5) or endosomes (TLR3, TLR7, TLR8, TLR9) are targets of adjuvants and include Pam3Cys (TLR2 ligand), Poly(I:C) (TLR3 ligand), monophosphoryl lipid A (MPL; TLR4 ligand), flagellin (TLR5 ligand), imiquimod (TLR7/8 ligand), and CpG oligodeoxynucleotides (ODNs; TLR9 ligand) [31]. When activated, TLRs recruit a group of adaptors, including myeloid differentiation factor 88 (MyD88), Toll-interleukin receptor (TIR) domain containing adaptor protein (TIRAP), TIR-domain-containing adaptor inducing interferon-β (TRIF), and TRIF-related adaptor molecule (TRAM), and then activate downstream signal transduction, thereby simultaneously activating the corresponding transcription factors, consequently leading to the secretion of chemokines and cytokines [32]. In the same way, adjuvants that target NLRs, such as muramyl dipeptide [33], and adjuvants that target RLRs, such as M8 [34], also activate the innate immune response and produce an immune-enhancing effect.

## Mechanism of self-adjuvanting nanovaccines for cancer therapy

Nanoparticles can have an immune-enhancing effect as adjuvants in nanovaccines for cancer therapy and improve the anti-tumor effect (Table 1). According to the mechanism underlying their self-adjuvanting properties, nanovaccines can be divided into four categories: (1) nanovaccines that enhance cross-presentation, which promote exogenous cancer antigens taken up by DCs and cross-presented for CD8^+^ T cell priming [35]; (2) nanovaccines that target the signaling pathways of the immune response; (3) nanovaccines that mimic desirable chemical and biological properties in nature; (4) nanovaccines with unknown mechanisms.

**Table 1:** Self-adjuvanting nanovaccines for cancer therapy

Self-adjuvanting nanovaccines for cancer therapy			
Nanovaccine	Immune modulation	Mechanism	Refs.
SPIO-OVA	IL-6, TNF-α, IFN-γ↑	Cross-presentation↑	[42–44]
α-Al2O3-OVA	CD8^+^T↑	Autophagy-related cross-presentation↑	[45]
γ-PGA-OVA	IgG2a, IgG2c, T cells, CTL↑	TLR4 and MyD88-dependent signaling pathway	[52–54]
VSSP-E7(p)	IFN-γ, IL-10, CD8^+^T↑	TLR4	[56, 58]
(R)-DOTAP-E7	IFN-γ, DC, CD4^+^T, CD8^+^T↑	TLR7, TLR9	[61, 62]
C1-mRNA	IL-1β, IL-6, IL-12, DC↑	TLR4	[63]
CPTEG: CPH/OVA	IgG1, IFN-γ, IL-12, DC, CD8^+^T↑	TLR2, TLR4, TLR5	[64–68]
3DSNA-OVA	IL-12, IL-6, CTL↑	Phosphorylation of IKK-αβ, IkB-α, and p65 in BMDC↑, NF-κB activation	[71]
LDH-OVA, pcDNA3-OVA/LDH(R1)	IgG1, IgG2a, INF-γ, CTL↑	NF-κB	[72–74]
Ag-PMIDA-CoO	IFN-γ, TNF-α, IL-12, IgG1, IgG2, MΦ, CD4^+^T, CTL↑	TNF-α↑, NF-κB	[75, 76]
ECPs-OVA	DC, CTL↑	MyD88-dependent NF-κB	[77]
HMS-OVA, DMOHS‐2S-OVA MSR-PEI: OVA	IL-1β, IFN-γ, IL-2, IL-4, IL-10, CD4^+^ and CD8^+^ effector memory T cells↑	NLRP3 inflammasome	[80–86]
AuNP-OVA	IL-1β, IL-18, TNF-α, IL-6, CD8 ^+^ T cells↑	NLRP3 inflammasome, NF-κB	[89–91]
PSM-OVA	IFN-I, TNF-α, IL-17a, DC↑, Th2↓	TRIF- and MAVS-dependent type I interferon secretion	[96]
PC7A-OVA	CTL, Th1, APCs↑	STING-dependent type I interferon secretion	[97, 99]
SeaMac	TNF-α, DC↑	STING	[100]
CNP-OVA Man-CTS-TCL	IgG, IL-2, IL-10, IFN-γ, NK↑	cGAS- and STING-dependent type I interferon secretion	[105–107]
PEI-4Bimi-OVA	IFN-I, DC, CTL↑	STING	[48]
VLPs	Ab, Th1, CTL, B cells ↑	Similar structure to viruses	[113]
CPMV, PVX, TMV, PapMV	IFN-γ, TNFα, M1, NK, DC, CD8^+^T↑	ssRNA, can activate TLR7/TL8	[118–122]
Archaeosome-OVA	DC, MΦ, CTL↑	Mimic the structure of microorganisms	[126]
Q11-MUC1, Q11-HPV16 E7 _44–62_	IgG2a, IgM, Th1, CTL↑, Th2↓	Unclear	[128–131]
CD-OVA	TNF-α, IFN-γ, DC↑	Unclear	[132]

### Enhancing cross-presentation

**Fig. 2:**
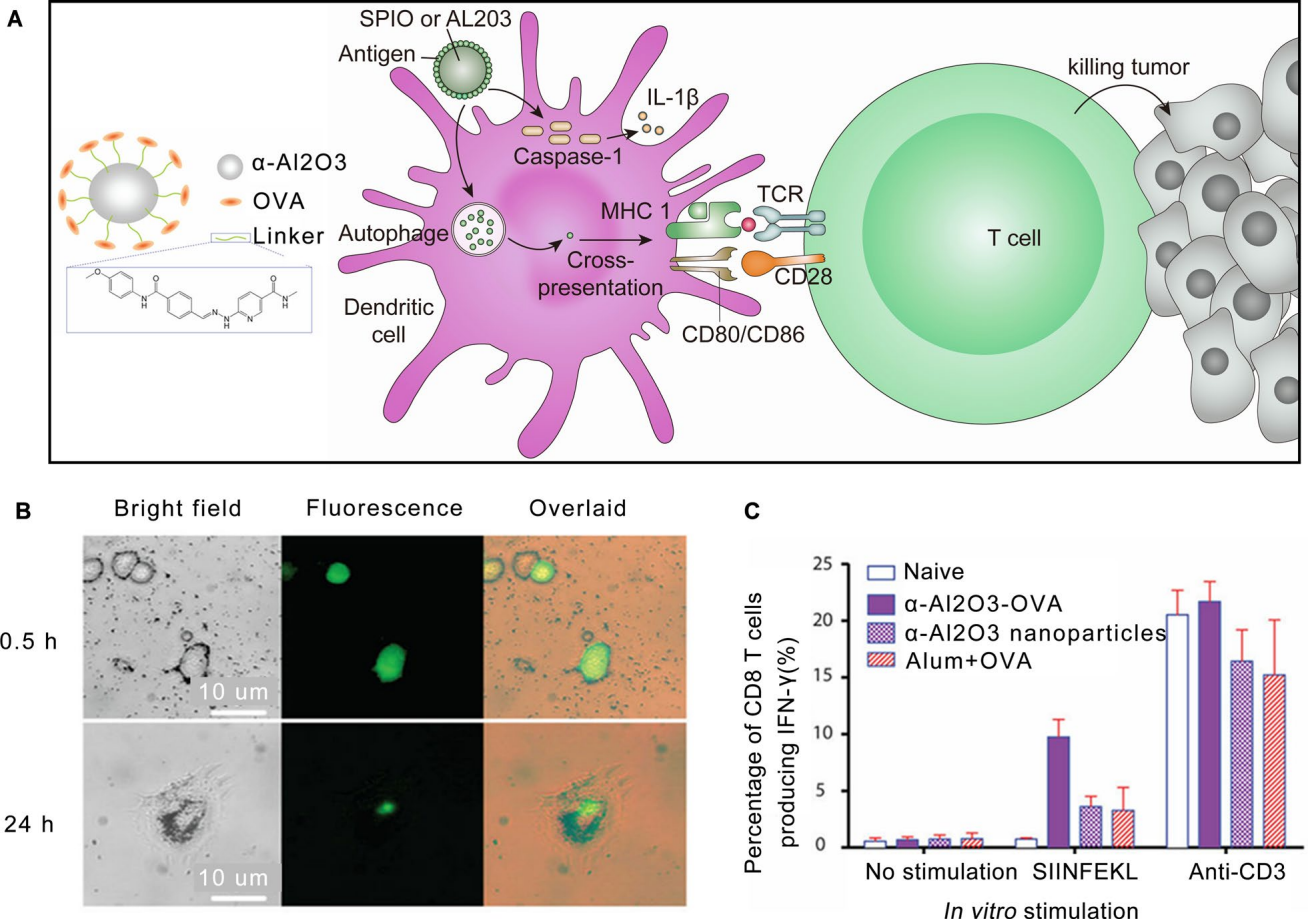
Enhancement of cross-presentation in dendritic cells by nanoparticles. **A** Schematic illustration of nanoparticles enhancing cross-presentation in dendritic cells. **B** Representative bright field (left), fluorescence (middle) and overlaid (right) images of DCs after incubation with FITC-labelled α-Al_2_O_3_ (60 nm)-OVA for 0.5 (upper) and 24 h (lower). **C** Vaccination with α-Al2O3-OVA induced high frequency of OVA-specific IFN-γ producing CD8^+^ T cells in spleens of mice [45]. Copyright 2011 Nature Publishing Group

The classical antigen presentation pathway is the presentation of endogenous antigens by MHC class I molecules to activate CD8^+^ T cells and the presentation of exogenous antigens by MHC class II molecules to activate CD4^+^ T cells [36]. Cross-presentation empowers DCs to bind foreign antigens to MHC class I molecules, thus activating CD8^+^ T cells [35]. Cross-presentation allows T cells to be activated in a diversified manner, orchestrating specific humoral and cellular immunity, and contributing to an anti-tumor immune response [37]. Effective cross-presentation is crucial for vaccination against cancer and infections caused by intracellular viruses and bacteria. Inspired by this strategy, a variety of nanovaccines has been developed to enhance cross-presentation and priming of CD8^+^ T cells [38]. Indeed, some self-adjuvanting nanovaccines for cancer therapy can enhance cross-presentation (Fig. 2A).

Superparamagnetic iron oxide nanoparticles (SPIO) are biocompatible and have been widely used in medical imaging and drug delivery [39, 40]. SPIO is a standard agent used in visualization research of vaccines, such as for the labeling of DCs to trace their migration to LN [41]. Recently, it has been reported that SPIO can enhance the intracellular delivery of antigens into APC by cross-presentation and that SPIO have the latent capacity to be a vaccine adjuvant [42]. Interestingly, in this study, positively charged SPIO enhanced cross-presentation, which resulted from increased cytosolic antigen delivery, while negatively charged SPIO inhibited the functions of DCs by autophagy [42]. In another study, Liu et al*.* found that the enhancement of cross-presentation by SPIO is related to IL-1β activity. Optimal IL-1β improved cross-presentation, while excess IL-1β induced by negatively charged SPIO inhibited this process [43]. SPIO-ovalbumin (OVA) nanovaccine, composed of OVA antigen and Fe_3_O_4_ nanoparticles, has the ability to promote the activation of APCs and the production of Th1 bias immune cytokines. These are secreted by macrophages and DCs, which significantly inhibits the growth of tumors after intratumoral injection compared with controls [44]. In this instance, SPIO serves as both a vaccine delivery system and an immune potentiator, providing a promising method to simplify the formulations of nanovaccines.

In addition to SPIO, Li et al. conjugated OVA to α-Al_2_O_3_ nanoparticles and discovered that DCs pulsed with α-Al_2_O_3_-OVA efficiently cross-presented OVA antigen to naïve OT-I T cells in vitro and in vivo [45] (Fig. 2B, C). Transmission electron microscopy (TEM) showed that this process is related to autophagy, and treatment with α-Al_2_O_3_-OVA led to tumor regression in tumor-bearing mice [45].

Autophagy in APCs has been reported to be related to cross-presentation [46]. However, due to the complexity of autophagy mechanisms and the diversity of autophagy substrates, the relationship between autophagy and cross-presentation is controversial [47]. In addition to inorganic nanovaccines, organic polymers like polyethylenimine (PEI-M) and biomimetic nanoparticles like virus-like particles (VLPs) can also play an adjuvant role by encouraging cross-presentation in cancer vaccines [48, 49]. It is worth noting that the specific mechanisms underlying the enhancement of cross-presentation by nanoparticles requires further elucidation and research.

### Targeting of signaling pathways

Nanovaccines for cancer therapy show a self-adjuvanting effect of targeting signaling pathways in the immune response, including targeting TLRs, NF-κB, NLRP3, and IFN-related signaling pathways (Fig. 1).

#### Targeting of TLR-related signaling pathways

Targeting TLRs is a common mechanism of adjuvants, and the self-adjuvanting effect of nanovaccines for cancer therapy is also related to this pattern recognition receptor.

**Fig. 3:**
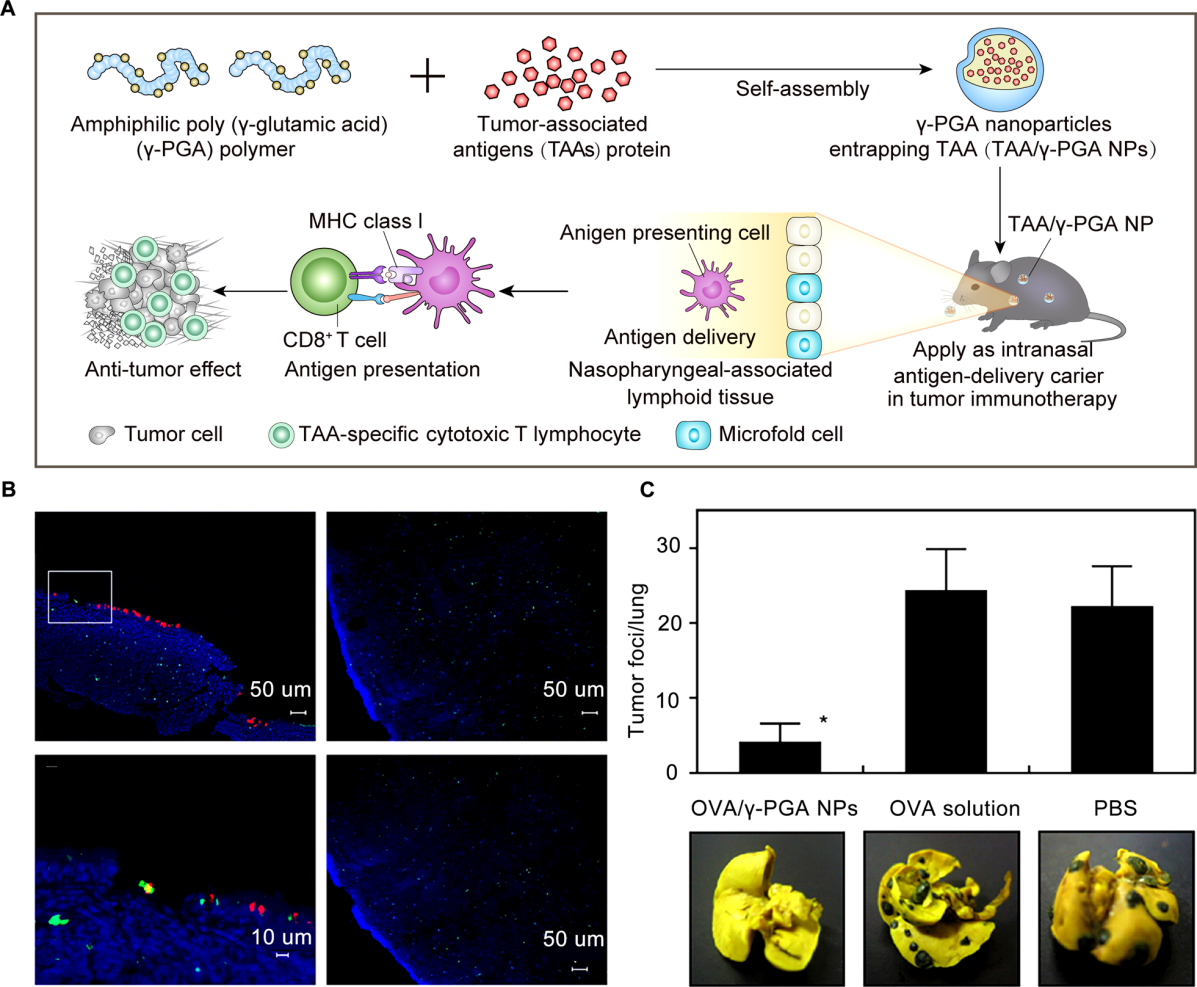
Potent tumor immunity induced by poly (γ-glutamic acid) nanovaccine via a TLR4 and MyD88 signaling pathway. **A** Schematic illustration of nasal vaccination with antigen-entrapping γ-PGA NPs evoked tumor immunity by eliciting antigen-specific CTLs. **B** Biodistribution of intranasally administered FITC-OVA/γ-PGA NPs. Green (FITC-OVA), red (rhodamine-labeled UEA-1), and blue (DAPI) signals were digitally merged. **C** Therapeutic effect of intranasal vaccination of OVA/γ-PGA NPs against B16-OVA lung metastasis. Reproduced with permission [54]. Copyright 2011 Elsevier B.V

Poly-γ-glutamic acid (γ-PGA) is a biocompatible polymer that is produced by culturing Bacillus licheniformis and Bacillus subtilis [50, 51]. Yoshikawa et al. demonstrated that γ-PGA nanoparticles could be used as antigen carriers in cancer vaccines [52]. Immunizing mice with OVA entrapped γ-PGA nanoparticles activated the CTL response and effectively delayed tumor growth in mice without toxic reaction. In addition to serving as a carrier, γ-PGA nanoparticles also activate APCs and induce a potent antigen-specific T cell response through the TLR4 and MyD88-dependent signaling pathway, elevating both innate and adaptive immune responses, especially cellular immunity [53]. Another profound virtue of γ-PGA nanoparticles is that the antigen-encapsulated nanovaccine can be vaccinated intranasally to induce antigen-specific CTL immunity, demonstrating the potential to manufacture non-invasive cancer vaccines in the future [54] (Fig. 3).

Similarly, very small size proteoliposomes (VSSP), formed by GM3 ganglioside and meningococcal outer membrane protein complex via hydrophobic interaction [55], also target TLRs to potentiate the immune response. Mesa et al. found that VSSP activated TLR4 on DCs, leading to an effective Th1 cell-mediated immune response [56]. Additionally, the activation of DCs by VSSP was observed on lipopolysaccharide (LPS)-hyporesponsive mice in this study, suggesting that other components of VSSP can also stimulate immunity. Furthermore, VSSP has a similar function as LPS but less toxicity and a better effect in humans than MPL-A. Therefore, VSSP may be a feasible agent to employ in DC activation [57]. The immune-potentiating property of VSSP also suggests that VSSP could be a potent vaccine adjuvant. Torréns et al. reported that vaccination with the E7 oncoprotein of human papillomavirus (HPV) type 16 and VSSP protects mice from tumor invasion, induces the established tumor regression, and produces an E7-specific CD8^+^ T cell response [58].

In the adjuvant mechanism of the cationic lipid 1,2-dioleoyl-3-trimethylammonium-propane (chloride salt) (DOTAP), it has been previously reported that DCs are activated through the extracellular-signal-regulated kinase (ERK) pathway [59]. Used as a vaccine adjuvant, an optimal dose of DOTAP combined with HPV16 E7-derived peptide inhibits TC-1 tumor growth [60]. Among the enantiomers of DOTAP, (R)-DOTAP is regarded as a more effective adjuvant than (S)-DOTAP in stimulating CD8^+^ T cells to secret interferon gamma (IFN-γ) against tumors [61]. The adjuvant mechanism of (R)-DOTAP is also related to TLRs, which targets TLR7 and TLR9 to induce the production of Myd88-dependent type I IFN, eventually leading to tumor regression [62]. Additionally, Zhang et al. developed a library of cationic lipid-like compound and found that C1 LNP with a 12-carbon tail effectively delivered antigen-encoding mRNA into DCs [63]. C1 LNP activated TLR4 on DCs and activated immunity while delivering mRNA. C1 LNP-formulated mRNA vaccine can be used as an effective tumor preventive and therapeutic cancer vaccine.

Furthermore, the adjuvant property of polyanhydride is also considered to be related to targeting TLRs. Polyanhydride, a biodegradable synthetic biopolymer explored for drug delivery, is facile to synthesize, is inexpensive and has been reported to activate TLR2, TLR4, and TLR5 on DCs, acting as an active Th1 adjuvant that efficiently trigger Th1 cell-mediated immune response [64, 65]. Wafa et al. designed a cancer vaccine, 20:80 1,8-bis-(p-carboxyphenoxy)-3,6-dioxaoctane (CPTEG):1,6-bis-(p-carboxyphenoxy) hexane (CPH) / OVA, which activated specific CD8 ^+^ T cells, produced specific IgG1 antibody, and prevented thymoma formation in mice subcutaneously challenged with a OVA-expressing thymoma cell line [66]. In a further study, they reported that a single vaccination dose of 20:80 CPTEG:CPH polyanhydride particles was sufficient to activate anti-tumor immunity [67]. Similarly, Darling et al. developed a prophylactic vaccine designed as a single-dose polyanhydride nanovaccine that activates DCs, induces antigen-specific CD8^+^ T cell memory, and reduces tumor burden [68].

#### Targeting of NF-κB related signaling pathways

The NF-κB signaling pathway is composed of the dimer transcription factor NF-κB/Rel, inhibitor IκB, and upstream IκB kinase IKK. Activating IKK phosphorylates IκB, then IκB is degraded, and NF-κB enters the nucleus to induce changes in gene expression [69]. This signaling pathway is closely related to inflammation and tumors [70]. The adjuvant effect of some nanoparticles in cancer vaccines is also related to this signaling pathway.

**Fig. 4:**
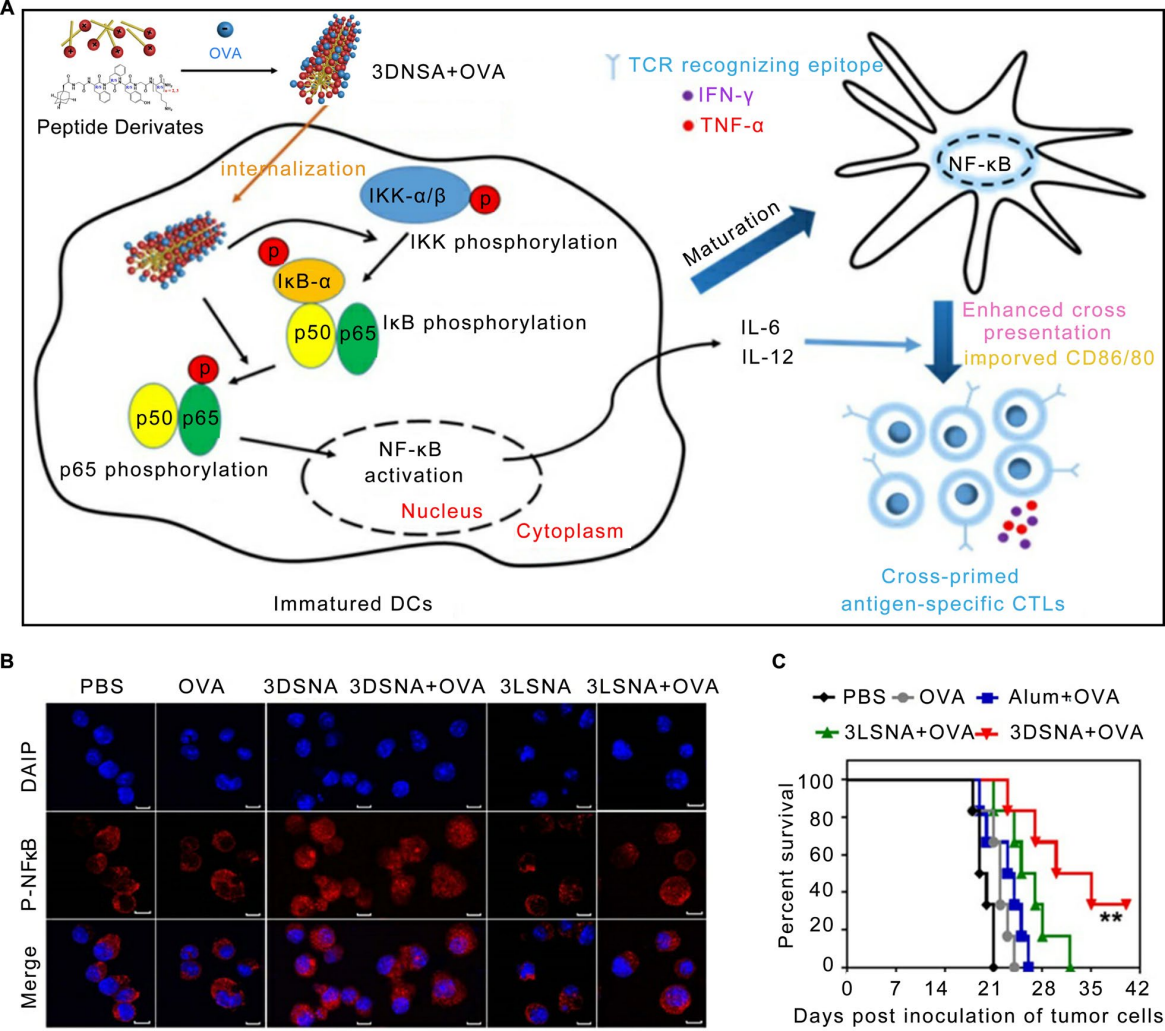
3DSNA nanovaccine activated the innate and specific immunity by the NF-κB signaling pathway. **A** Schematic of 3DSNA as versatile adjuvants that initiate antigen-specific CTL responses for cancer immunotherapy. **B** The analysis of p-p65 by laser scanning confocal microscopy. **C** The survival of tumor-bearing mice treated with different formulations after tumor challenge. Reproduced with permission [71]. Copyright 2019 Ivyspring International Publisher

One such adjuvant is 3DSNA, a supramolecular nano-adjuvant that self-assembles from positively charged D-peptide derivatives [71]. A nanovaccine fabricated with 3DSNA and OVA effectively enhances the phosphorylation of IKK-αβ, IkB-α, and p65 in BMDCs, then activates the NF-κB signaling pathway, and eventually enhances the immune response. This vaccine has both preventive and protective effects on tumors in situ [71] (Fig. 4). Despite this, upstream targets of the NF-κB remain unknown and require more research.

Layered hydroxide (LDH) nanoparticles, formulated with different ratios of Mg^2+^ and Al^3+^, can activate DCs possibly by increasing the expression of NF-κB in the nucleus in a dose-dependent manner and promote the reduction of total IκBα levels [72]. This pathway is related to uptake of LDH by DCs, induction of DC maturation and CCR7 upregulation. LDH nanomaterials in the form of nanoparticles or nanosheets as adjuvants of cancer vaccines have a significant effect on inhibiting tumor growth [73, 74].

In addition, the adjuvant effect of cobalt oxide (CoO) nanoparticles in cancer vaccines is also believed to be related to the NF-κB signaling pathway. Developed by Chattopadhyay et al. [75, 76], CL (human oral cancer cell lysate)-PMIDA (N-phosnomethyliminodiacetic acid)-CoO nanoparticles deliver tumor lysates to macrophages. Subsequently, they induce the release of TNF-α, activate the NF-κB signaling pathway, and exert anti-tumor effects both in vitro and in vivo. Future studies may expand the application of this adjuvant to other tumor models.

Su et al. developed a co-assembled hydrogel vaccine which was co-assembled by supramolecular antigen epitope-conjugated peptides (ECPs) targeting CD8 or CD4 T-cell receptors [77]. The co-assembled peptide hydrogel vaccine effectively activated the MyD88-dependent NF-κB signaling pathway in DCs, displaying superior antitumor effect than Alum-adjuvanted epitope vaccine in E.G7-OVA tumor model.

#### Targeting of NLRP3 inflammasome related signaling pathways

Inflammasomes are protein complexes composed of NLRs and melanoma 2 (AIM2)-like receptors (ALRs), which activate the pro-inflammatory factors caspase-1 and caspase-11 [78]. NLRP3 inflammasome, the most widely studied inflammasome, is closely related to the occurrence of infection and tumors [79, 80].

**Fig. 5:**
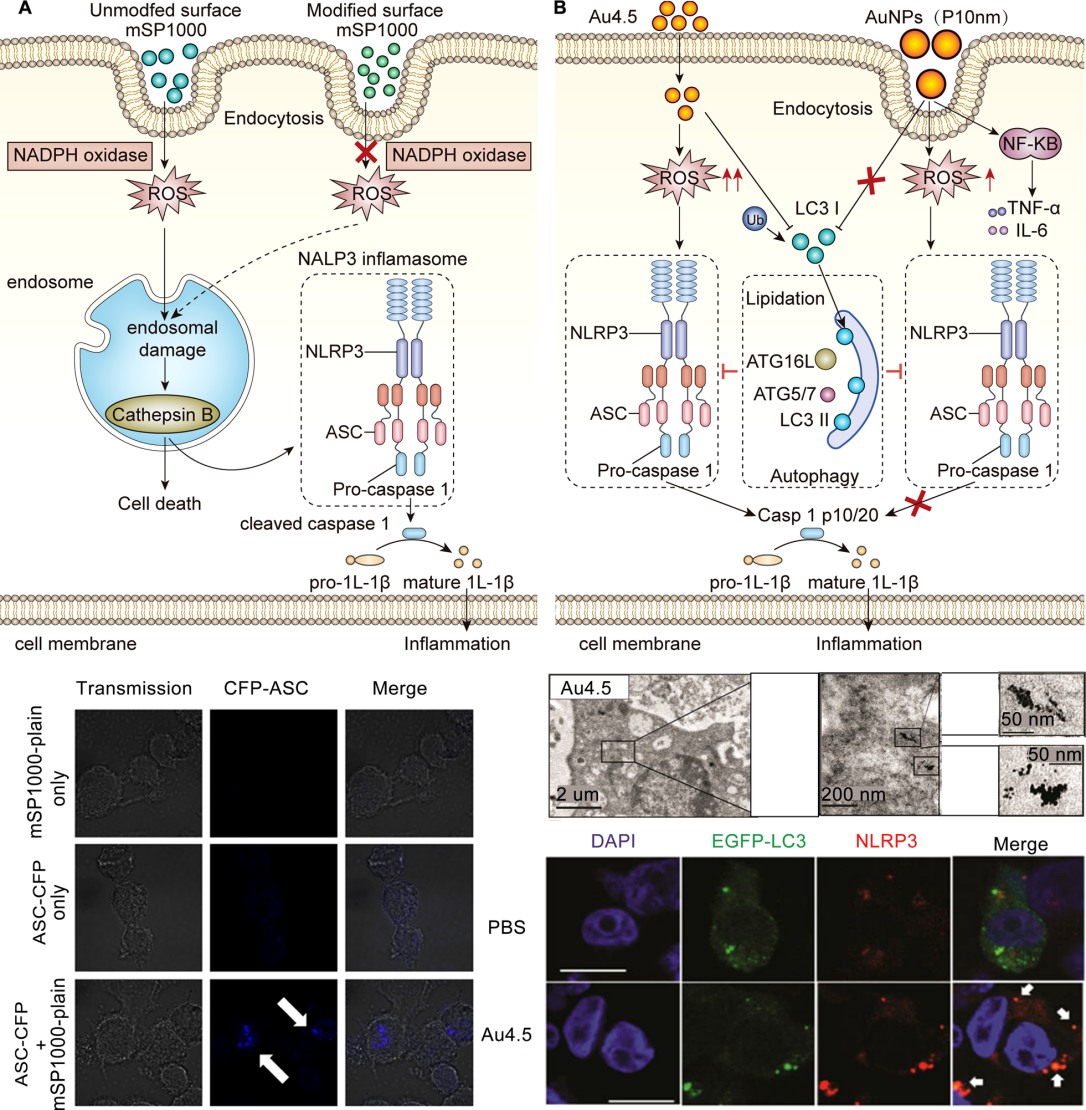
Adjuvants activated the NLRP3 inflammasome to improve the ability of nanovaccines to induce immune responses. **A** Model of mSP1000-induced IL-1β maturation via assembly of NALP3 inflammasomes. Reproduced with permission [81]. Copyright 2010 Elsevier Ltd. **B** Schematic illustration of Au4.5-induced NLRP3 inflammasome activation. Reproduced with permission [91]. Copyright 2020 American Chemical Society

The adjuvant effect of silica particles was found to be related to the NLRP3 inflammasome in APCs. Amorphous silica particles can activate NADPH oxidase, leading to ROS production, endosomal rupture, cathepsin B leakage to the cytoplasm, and NLRP3 inflammasome assembly on THP-1 macrophage-like cells, thereby inducing IL-1β production [81] (Fig. 5A). Furthermore, NLRP3 inflammasomes in DCs play a key role in stimulating the production of IL-1β and regulating the infiltration of immune cells by mesoporous silica microrods (MSRs) [82]. Different structures of mesoporous silica nanoparticles (MSNs) were designed as a versatile platform for cancer vaccines to both deliver antigens and amplify antigen-specific immune responses, such as hollow mesoporous silica (HMS) nanospheres, double‐shelled dendritic mesoporous organosilica hollow spheres (DMOHS‐2S), and MSR [83–86].

Gold nanoparticles provide strong optical properties and unique surface plasmon resonance (SPR) properties and have been used in optical imaging, immunoassays, drug delivery, and other fields [87, 88]. Gold nanoparticles are considered a versatile tool in the diagnosis and treatment of tumors [88]. Almeida et al. found that AuNP-OVA particles promoted an antigen-specific immune response and exerted anti-tumor effects in both preventive and therapeutic models, which suggested that gold nanoparticles could be used as adjuvants with superior immune stimulation [89]. Importantly, this immune-stimulating effect of gold nanoparticles depends on the nanoparticle size and shape [90]. In 2020, a study by Zhu demonstrated that ultrasmall (4.5 nm) gold nanoparticles triggered the ROS production and targeted the microtubule-associated protein 1 light chain 3B (LC3) to eventually activate the NLRP3 inflammasome in DCs, thus enhancing antibody production [91] (Fig. 5B). This study also showed that large gold nanoparticles (3, 30, and 70 nm) triggered the NF-kB signaling pathway [91], but no further study was conducted.

In addition, there are also organic polymers that can activate NLRP3 inflammasome related signaling pathways. For example, Manna et al. developed a minimal activation system using only a short peptide coupled with an ethylene glycol sequence to activate the NLRP3 inflammasomes in DCs [92]. The NLRP3 inflammasomes may also be triggered by dendronized polypeptides (denpols) and eventually enhance cross-presentation [93]. The self-adjuvanting properties of these organic polymers suggest their potential for the development of cancer nanovaccines.

#### Targeting of IFN-related signaling pathways

IFN is a cytokine with significant anti-tumor and immunomodulatory effects [94]. Some nanoparticles exhibit a self-adjuvanting effect in cancer vaccines through IFN-related signaling pathways.

Porous silicon nanoparticles, as nanoparticles with controllable geometry, adjustable nanostructures, and a variety of surface chemical properties, have been increasingly applied in the field of drug delivery and cancer immunotherapy [95]. Cancer vaccine based on porous silicon can be phagocytosed by DCs and induce DCs to secrete TRIF- and MAVS-dependent type I IFN, resulting in strong anti-tumor efficacy [96].

**Fig. 6:**
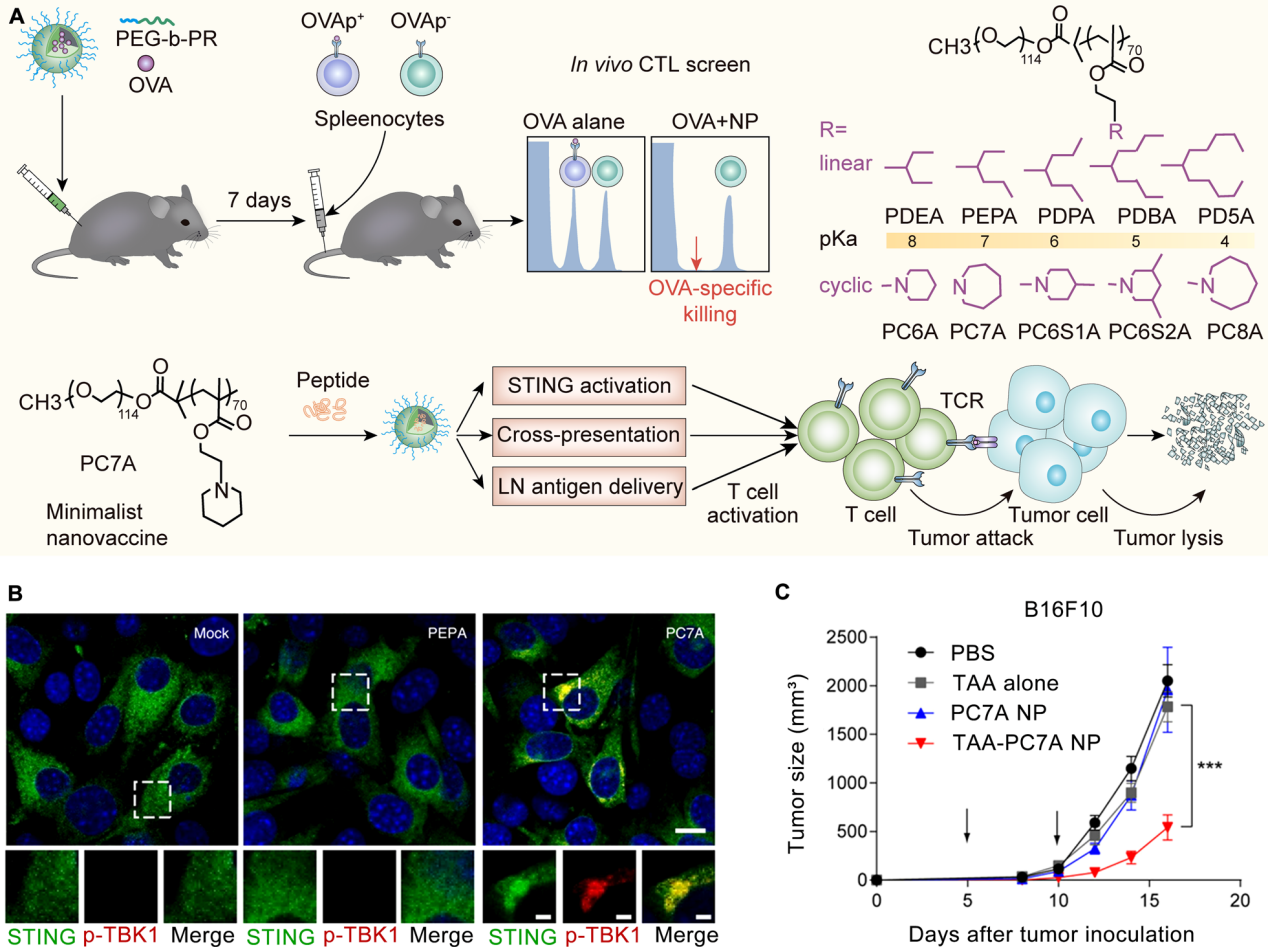
PC7A nanovaccine activated the STING pathway and inhibited tumor growth. **A** Schematic of the design and mechanism of the PC7A nanovaccine. **B** p-TBK1 is recruited into the STING–PC7A condensates. Reproduced with permission [98]. Copyright 2021 The Author(s), under exclusive licence to Springer Nature Limited. **C** Tumor growth inhibition study of B16F10 melanoma [97]. Copyright 2017 Nature Publishing Group

Moreover, PC7A nanoparticles were screened in vivo from an ultra-pH sensitive (UPS) nanoparticle library developed by Luo’s laboratory. These nanoparticles have the ability to induce specific CTL and Th1 responses and promote type I IFN secretion by stimulating the STING pathway [97, 98]. Luo et al. also reported a synergistic effect between the PC7A nanovaccine and radiotherapy [99]. Self-adjuvantinged molecular activator (SeaMac) nanovaccines constructed by PC7A nanoparticles significantly inhibited tumor growth in CT26 and B16-F10 tumor models [100] (Fig. 6).

Chitosan, a linear polysaccharide composed of β-(1,4)-linked N-acetyl-D-glucosamine units, is a bioactive polymer with multiple applications in wound healing [101], antibiosis [102], and drug delivery [103]. Lin et al. cultivated monocytes in chitosan substrate and found that chitosan induced the differentiation of monocytes into DCs [104]. On this basis, vaccination of DCs pulsed by tumor lysate demonstrated a strong anti-tumor effect. Chitosan nanoparticles also have the capability to promote both cellular and humoral immune responses [105]. Furthermore, Carroll et al. found that chitosan promoted the cellular immunity by activating cGAS-STING in DCs, leading to type I IFN-dependent DC activation [106]. Based on the above studies, it can be inferred that chitosan nanoparticles may be a promising vaccine adjuvant. Shi et al. developed mannose(Man)-chitosan-tumor cell lysate (TCL) nanoparticles as cancer vaccine, which displayed a significant anti-tumor effect in vitro and in vivo [107].

In addition, Zhao et al. constructed a series of azole molecules end-capped PEI-M which could activate the STING pathway and induce type I IFN secretion from DCs [48]. Further, they designed a minimalist binary nanovaccine (BiVax) consisted of OVA and PEI-4BImi, and the BiVax showed better performance than both traditional PEI/cGAMP/antigen ternary vaccine and Alum adjuvant-based vaccine in antitumor activity in B16-OVA tumor-bearing mice. Interestingly, the BiVax composed of antigens from resected tumor tissues with PEI-4BImi inhibited the recurrence of postoperative MC38 tumor effectively, which displayed the great prospect of personalized vaccine.

### Biomimicking the natural invasion process of pathogens

A biomimetic design is another way to enhance the vaccine efficacy of nanoparticles. Simulating natural infections can activate innate immune responses through via pattern recognition receptors (PRRs), which contribute to generate lasting adaptive immunity [108]. Cancer vaccines based on recombinant plant viruses, virus-like particles, and archaeosomes use this method to exert a self-adjuvanting effect.

#### Biomimicking the structure of virus

**Fig. 7:**
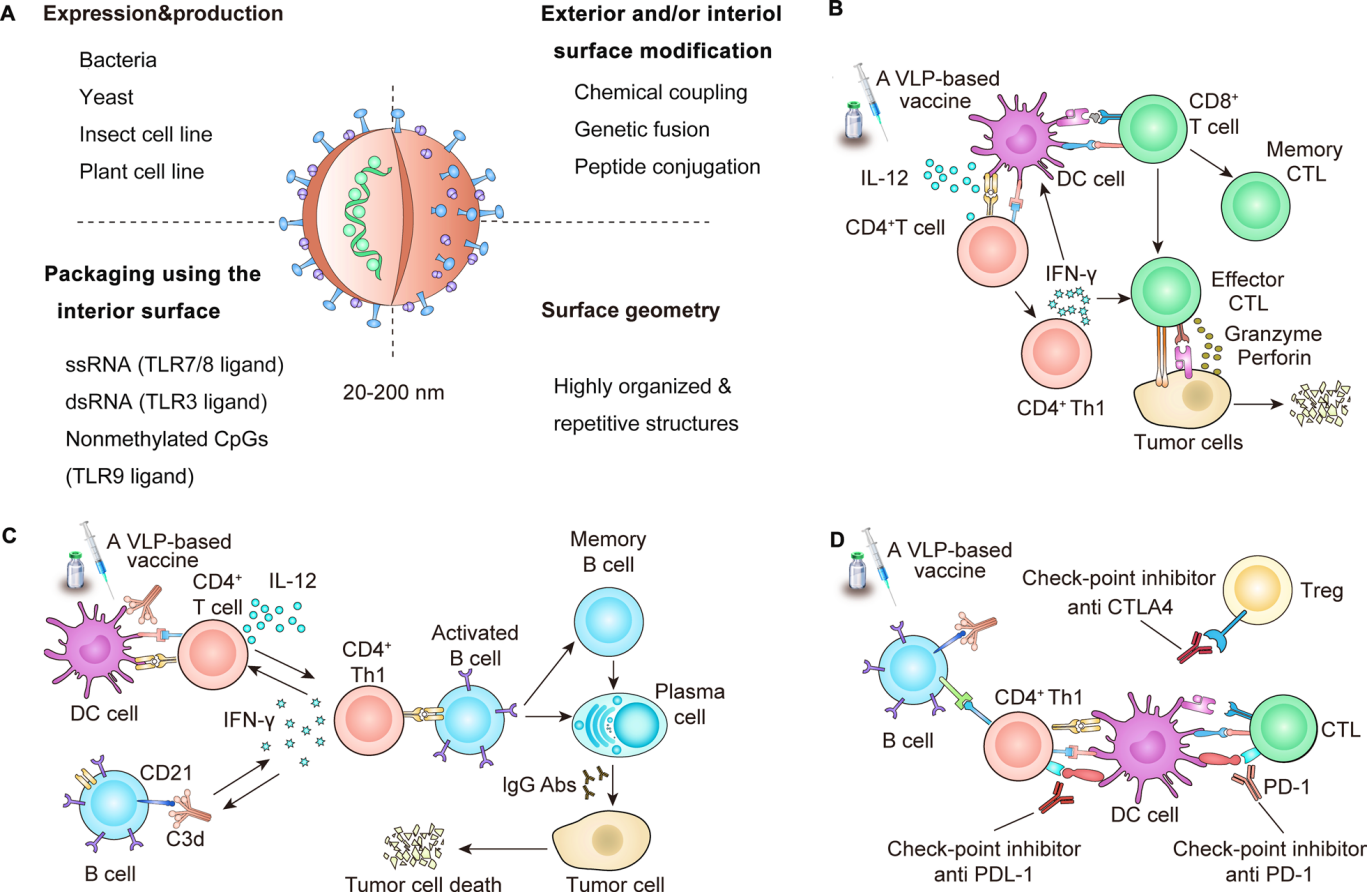
self-adjuvanting effect of VLPs in cancer vaccines. **A** Key characteristics of VLPs. **B** T cell responses induced by VLP-based vaccines. **C** B cell responses induced by VLP-based vaccines. **D** Vaccines in the context of checkpoint inhibitors. Reproduced with permission [113]. Copyright 2020 The Author(s)

VLPs are formed by the self-assembly of envelope and/or capsid proteins from many viruses. VLPs have a similar structure to viruses, but lack the viral genome and therefore cannot replicate [109]. VLPs could be a safe and versatile platform for vaccines due to their capacity of rapid drainage to LNs, efficient antigen display, and effective delivery of adjuvants [110]. VLPs have a unique repetitive surface structure with an effective pathogen associated structural pattern (PASP) that promotes cross-linking with B cell receptors [111]. VLPs are effectively taken up by APCs, especially by DCs, and thus can be regarded as an exogenous antigen to be presented by MHC class II molecules, which can also combine with MHC class I molecules by cross-presentation to activate humoral and cellular immunity [112]. At present, VLP-based vaccines are a promising strategy for cancer treatment, which has been discussed in melanoma, breast cancer, pancreatic cancer, cervical cancer, hepatocellular carcinoma, and other tumors [113] (Fig. 7).

The unique properties of plant viruses, including their ability to self-assemble and their biosafety for mammals and humans, make them an attractive and versatile tool in biotechnology [114]. Recombinant plant viruses with simple compositions, such as cowpea mosaic virus (CPMV), potato virus X (PVX), papaya mosaic virus (PapMV), and tobacco mosaic virus (TMV), can induce humoral and cellular immunity and are possible vaccine candidates [115]. Distinct from VLPs, recombinant plant viruses contain ssRNA that activates TLR7/TL8 and induce NF-κB, IRF, and other signaling pathways to stimulate immune responses [116]. The complement system is also believed to be involved in the recognition and phagocytosis of PapMV and regulate the production of IFN-α after TLR7 activation [117]. Nanovaccines for cancer therapy based on CPMV, PVX, PapMV, and TMV have shown impressive effects in animal experiments [118–123]. Furthermore, Shukla et al. prepared CPMV and eCPMV in situ vaccines and found that intraperitoneally administered CPMV gave rise to an antibody response. Moreover, this study demonstrated that prior exposure to CPMV enhanced the efficacy of CPMV in situ vaccine for ovarian cancer, and the same holds true for eCPMV. This idea of using recombinant plant viruses to activate the inherent ability of innate immunity to improve the anti-tumor effects holds promise, but specific molecular mechanism and reasonable applications remain to be further explored.

#### Biomimicking the structure of archaea

Archaeosomes are liposomes formulated with ether glycerolipids extracted from various archaea. They have strong adjuvant properties and mimic the structure of microorganisms to provide danger signals of infection and accordingly activate both humoral and cellular immunity [124]. The strong adjuvant properties of archaeosomes are also believed to be related to the enhancement of cross-presentation of antigens and the improvement of the immunomodulatory ability of APCs [125, 126]. Developed by Krishnan, OVA-archaeosome vaccination protected mice from the development of EG.7 solid tumor cells, and this immune effect is mediated by CD8^+^ T cells [127].

### Nanovaccines with other mechanisms

Some nanovaccines for cancer therapy possess self-adjuvanting properties, but their mechanisms remain not fully clear and warrant further investigation.

#### Q11 peptide

Q11 (Ac-QQKFQFQFEQQ-Am) is a designed short peptide that can self-assemble in an aqueous environment to form β-sheet rich nanofibers and displays functional amino acid sequences or chemical groups on the surface of its self-assembled fibers [128]. In addition, Q11 can be used as a carrier or an adjuvant, and its adjuvant properties depend on its covalent binding to the epitope. Cancer vaccines containing a variable number of full-length tandem repeat domains of MUC1 and Q11 can trigger a significant immune response, including complement-dependent cytotoxicity against MCF-7 cells [129]. Nanofibers prepared by chemically linking the HPV16 E7 peptide and the N-terminus of the self-assembling peptide Q11 also prevent the formation of transplanted TC-1 tumors and suppress the growth of established TC-1 tumors [130]. Similarly, peptide Coil29 (QARILEADAEILRAYARILEAHAEILRAD), a peptide composed of almost complete α-helical structures, also induces strong humoral and cellular immune responses without adjuvant [131]. Furthermore, Wu et al. compared the immune responses raised by Q11 and Coil29, and found that Coil29 evoked antibody responses with a higher titer and quality because of better germinal center B cell formation, better acquisition and activation of DCs, and better Tfh cell responses [131]. This nanofibers-forming self-assembled peptide system can be used as a promising vaccine platform, but its mechanism for immunomodulation remains unclear.

#### Fluorescent carbon dots (CDs)

CDs are zero-dimensional nanocarriers with a diameter of less than 10 nm. They have the advantages of dispersibility, low toxicity, biocompatibility, biodegradability, wide raw material sources, and low cost. Hence, CDs are used in biological imaging, biological detection, and cancer treatment [132]. On account of the non-toxicity of PEG-modified CDs, Luo et al. designed a nanovaccine composed of CDs with PEG surface passivation and the model tumor antigen OVA, which positively contributes to antigen uptake, efficiently accelerates the maturation of DCs, stimulates splenocyte proliferation, induces the production of IFN-γ, and eventually inhibits the growth of established B16 melanoma tumors expressing OVA [133]. Due to spectral effects, fluorescent CDs are expected to be an effective immune adjuvant to enhance cancer immunotherapy, but the specific mechanism requires further study.

To summarize, we have classified self-adjuvanting cancer nanovaccines according to their underlying immune mechanism. Enhancing cross-presentation activates cell-mediated immunity and can induce a robust antigen-specific CTL response, both key components of antitumor immunity. However, in some cases, it is not sufficient to enhance cross presentation alone to activate systemic anti-tumor immunity and tumor-associated DCs could be impaired by the inhibitory factors in tumor microenvironment [134]. On the other hand, targeting signaling pathways in the immune response promotes DC maturation, triggers cascade release of proinflammatory cytokine, and boosts innate and adaptive immunity. Nevertheless, there is a possibility of systemic toxicity if overactivated [135]. Moreover, biomimicking of the natural invasion process of pathogens activates both humoral immunity and cellular immunity, and the variety of VLPs and archaea makes them structurally attractive and functionally diverse. In terms of cancer treatment and prevention, more evidence is needed regarding the potential efficacy, side effects and benefits of VLPs and archaea based vaccines [136]. In addition, a variety of factors can influence the immune activation mechanism of nanoparticles, such as particle size, charge, and surface modification. For example, cationic nanoparticles facilitated better endosomal escape and higher cross-presentation [42], and amorphous silica with sizes ranging from 70 to 100 nm facilitated endosomal escape [137]. Different microorganism exposure during phylogeny may also affect the selection of recognition receptors [136]. This could account for differences in recognition receptors between species. It is worth noting that nanoparticles frequently could activate immunity through more than one mechanism. An identification of the mechanism can be performed by determining whether the nanoparticles have bionic structure, detecting the expression of costimulatory molecules and cytokine secretion by DCs after incubation with nanoparticles in vitro, and observing the changes of tumor immune microenvironment and systemic immunity after nanovaccines administration in vivo.

## Self-adjuvanting nanovaccines for cancer therapy in clinical research

At present, some clinical studies have provided convincing evidence that self-adjuvanting nanovaccines are a promising strategy for cancer therapy. VSSP-based self-adjuvanting nanovaccines are under investigation in clinical trials in patients with breast cancer, prostate cancer, high-grade cervical intraepithelial neoplasia and other solid tumors [138–142]. Caballero et al. developed a cancer vaccine based on the extracellular domain of HER1 (HER1-ECD) using VSSP and Montanide ISA 51 as adjuvants [139]. In their phase I study trial in 24 prostate castration-resistant carcinoma patients, the HER1 vaccine was shown to be safe and immunogenic [139]. A 10-year follow-up of patients vaccinated with another prostate cancer vaccine based on VSSP also reported a positive impact of vaccination on overall patient survival compared with those receiving the standard treatment [142]. Similarly, CIGB-247, a therapeutic cancer vaccine composed of recombinant modified human vascular endothelial growth factor (VEGF) and VSSP, is considered safe, tolerable, and immunogenic, as has been supported by their phase I clinical trial [140]. Furthermore, early clinical studies of VLPs as immunopotentiators in cancer vaccines have been carried out in different solid tumors, and the trails in melanoma and cervical intraepithelial neoplasia have provided promising results [113]. However, clinical studies on self-adjuvanting nanovaccines for cancer therapy are still in their infancy and need larger and deeper research in the future. More kinds of nanovaccines with self-adjuvanting properties should be included in the clinical research.

## Advantages of self-adjuvanting nanovaccines for cancer therapy

Some self-adjuvanting nanovaccines have entered early clinical research and showed satisfactory safety and effect, as mentioned above. Indeed, the self-adjuvanting properties of nanovaccines for cancer therapy have some unique advantages, which are discussed below.

### Simplification of vaccine composition

As vaccine development is directed toward “minimal” compositions, to focus the immune response on the target antigens [143], there is an urgent need to develop vaccines with both maximum efficacy and simplicity. Nanoparticles can be used simultaneously as vaccine delivery systems and immune enhancers, which simplifies the vaccine composition and avoids unnecessary side effects. As an example described above, the minimalist nanovaccine composed of antigen and synthetic polymer nanoparticles PC7A produces a strong cytotoxic T cell response combined with low systemic cytokine expression [97]. BiVax based of PEI-4BImi also outperforms both traditional ternary nanovaccines and commercially available aluminum-based vaccine [48]. DOTAP/E7 complex is another therapeutic cancer vaccine, consisting of only antigens and cationic liposomes, which can be absorbed by DCs to induce antigen-specific CTLs, and exhibit anti-TC-1 tumor effects [60]. Due to its simple composition, the cost of vaccine production may be reduced and the controllability for vaccine preparation may be increased [144]. As mentioned above, self-adjuvanting nanofibrous peptide hydrogel can be prepared by supramolecular peptide co-assembly [77]. Compared with traditional free peptide vaccine and Alum-adjuvanted vaccine, this self-adjuvanting nanovaccine induced strongest T cell response, and can be extended to vaccines targeting neo-epitope. Furthermore, Aiga et al. demonstrated that surface chemical modification was not necessary due to the inherent immune activity of the self-adjuvanting nanovaccine, which further simplifies the vaccine synthesis [145].

### Enhancement of the effects of other adjuvants

If a nanomaterial platform delivers adjuvants, it can also enhance the effects of other adjuvants. The combination of stellated fibrous mesoporous silica nanospheres and poly(I:C) (a synthetic double-stranded ribonucleic acid (dsRNA) analogue and immune enhancer) significantly reduced the dosage of poly(I:C) required in cancer vaccines for stimulating anti-tumor immunity [146]. In addition, co-delivery of CpG and OVA by the nanomaterial MgAl-(LDH) also induced higher levels of IgG1 and IgG2a antibodies, and the delivery by LDH induced a shift in the immune response from Th2 to Th1 [147]. Furthermore, Xu et al. designed a pathogen-like polymeric system comprising mannan-decorated nanoparticles as a TLR4 agonist that could synergize with CpG for maximally activating DCs [148].

### Improvement of the safety of vaccines

Over decades, many adjuvants have been proposed for vaccine development, but few have been widely applied due to their toxicity [149]. Self-adjuvanting nanovaccines alleviate this concern. Safety and stability have been improved in self-adjuvanting nanovaccines made by biocompatible nanoparticles, offering a simple, safe and robust strategy for boosting anti-tumor immunity for cancer therapy [60]. Furthermore, using adjuvants in VLPs vaccine formulations may increase the immunogenicity of the vaccine and stimulate specific type of immune responses [136]. Compared with vaccines admixed with traditional adjuvants, self-adjuvanting vaccines provide some advantages: (1) While achieving the same immune function these can greatly reduce the dosage of adjuvant, or even do without it. (2) These can provide more physical space for the loading of antigens. (3) By loading adjuvants at the same time, self-adjuvanting vaccines can also trigger the systemic immune responses from different mechanisms, which is expected to form an efficient and synergistic activation mode. The mannan-decorated pathogen-like polymeric nanoparticle system mentioned above could be an example.

## Challenges of self-adjuvanting nanovaccines for cancer therapy

Only a few self-adjuvanting nanovaccines for cancer therapy have started clinical research, as some challenges have to be overcome in the development of vaccines. (1) The mechanisms of the intrinsic immunopotentiation of different nanoparticles in cancer vaccines have not been fully elucidated and require more fundamental investigation. (2) Nanoparticles with immunostimulatory effect can also indirectly induce many immunotoxic effects. For example, nanoparticles entering the circulation may cause activation of complement cascade and antibody response, leading to undesirable side effects, including inflammation and allergic reactions [150]. (3) The immunostimulatory effect of nanoparticles with self-adjuvanting properties is complex and involves multiple signaling pathways, and the recognition and interaction between nanovaccines and immune cells has not been well identified. With the development of single-cell sequencing technology, the interaction between nanoparticles and specific APCs may be elucidated. Furthermore, more accurate delivery of nanovaccines to specific subtypes of APCs becomes possible, which will contribute to precisely manipulate the subsequent immunological responses. Additionally, in the current research of self-adjuvanting nanovaccines, more attention is paid to T cell immunity. Research have shown that VLPs can directly activate antigen-specific B cell and enhance humoral immunity through B cell-intrinsic MyD88 signaling [151]. However, there is still a lot of confusion about whether B cells, NK cells and other immune cells are affected, and how immunostimulatory nanoparticles affect them. (4) The ‘rules of immunogenicity’, or how the immune system responds to a given adjuvant or vaccine, depend greatly on the environment. As we know, nanovaccines injected intradermally or subcutaneously passively drain to LN through afferent lymphatic vessels, and then enter the subcapsular sinus to be taken up and processed by resident APCs [152]. During tumor development, the lymphatic microenvironment has been affected to some extent. For example, tumor-draining LNs undergo massive remodeling, including accumulation of immunosuppressive cells, reprogramming of stromal cells and vascular remodeling [153–155]. Leary et al. found that extracellular vesicles derived from melanoma cells selectively interact with LN resident macrophages and lymphatic endothelial cells, induce LN remodeling and eventually impair anti-tumor immunity [156]. Therefore, how to adapt to the changing lymphatic microenvironment should be considered in vaccine development. Besides, the distribution of nanovaccines in the subareas of LN also influences the strength of induced immune response [157]. It was reported that subcapsular sinus (SSC) macrophages prevented nanovaccines from accessing LN follicle. Depleting SCS macrophages increased the neutralized antibody production, showing the effective activation of humoral immunity [158]. What’s more, the vaccine response is directed by precise spatio-temporal cues [159], so how to reasonably design the self-adjuvanting nanovaccines to control the interaction between vaccine components and immune cells spatially and temporally also requires attention. (5) Scaling up nanovaccines is difficult, and clinical translation is a time-consuming process. As previously stated, only a handful of self-adjuvanting nanovaccines for cancer therapy have entered early clinical trials in patients with several types of solid tumors with preliminarily validation of safety. To better explore the efficacy and safety of emerging cancer nanovaccines, preclinical models related to human tumor development and its complex tumor microenvironment are urgently needed. Personalized medicine is also still a field in its infancy, and continued research along these lines will undoubtedly lead to better treatment options for patients in the clinic. These limitations mentioned above have resulted in a lack of significant investment in the development of the self-adjuvanting nanovaccines. However, these demerits may be subjugated with appropriate ideas and further advances in biotechniques and material science.

## Summary

In this review, we discussed the emerging class of nanovaccines; self-adjuvanting nanovaccines, and summarized the mechanisms of their self-adjuvanting properties, including the enhancement of cross-presentation, targeting of signaling pathways in the immune response, and biomimicking of the natural invasion process of pathogens. The mechanisms of certain nanoparticles with intrinsic immunomodulatory effect remain unclear and need to be further studied. To achieve robust antitumor T cell responses in cancer nanovaccines, it is imperative to orchestrate antigen cross-presentation with innate stimulation of APCs spatiotemporally. The release of antigens to the cytosol is a necessary step for antigen cross-presentation to CD8^+^ T cells, whereas innate stimulation mostly takes place in the endosomes since the PRRs for the commonly used adjuvants are mostly endosome receptors. Nanoparticles with multiple self-adjuvanting properties may be rendered the ability to maximally overcome the obstacle for intracellular coordination of APC activation and antigen cross-presentation to CD8^+^ cytotoxic T lymphocytes. Some self-adjuvanting nanovaccines for cancer therapy are in the early stage of clinical research and require larger and more in-depth studies. Moreover, we discussed the advantages and challenges of self-adjuvanting nanovaccines in cancer therapy. From what has been discussed above, we may reasonably arrive at the conclusion that it will provide a research direction to ameliorate the design of cancer vaccines if the self-adjuvanting property of nanovaccines is employed properly. A great deal of research remains to be done on the mechanism of self-adjuvanting nanovaccines, their intricate interactions, and practical application in cancer therapy. In addition to DCs and SSC mentioned in the article, the interaction between nanoparticles and other immune cells also deserves attention.
